# Why do psychiatric patients attend or not attend treatment groups in the community: A qualitative study

**DOI:** 10.1371/journal.pone.0208448

**Published:** 2018-12-13

**Authors:** Merve Dilgul, Philip McNamee, Stavros Orfanos, Catherine Elizabeth Carr, Stefan Priebe

**Affiliations:** 1 Unit for Social and Community Psychiatry, Blizard Institute, Queen Mary University of London, London, United Kingdom; 2 East London NHS Foundation Trust, London, United Kingdom; Teesside University, UNITED KINGDOM

## Abstract

**Background:**

Non-attendance of treatment groups in the community has been a long-standing problem in mental health care. It has been found to have financial ramifications for services, worsen outcomes for those that do not attend and negatively impact on therapeutic group processes. There is a need to gain a better understanding of patients’ reasons for attending or not attending. This study aimed to explore patient views on facilitators and barriers to the attendance of treatment groups in the community.

**Methods:**

The study used interview data collected as part of three studies that investigated treatment groups for psychiatric patients in the community. Sixty-seven interview transcripts were analysed using the framework method.

**Results:**

Five themes relating to facilitators of group attendance were identified: opportunity for autonomy; self-acknowledging need and therapist encouragement; optimal group format and safe environment; interest in content and enjoyment; actual and expected benefits of attendance. Four themes related to barriers: not being sufficiently informed; concerns about social interactions and the unknown; limited accessibility; and negative group dynamics.

**Conclusion:**

To facilitate attendance and reduce attrition to treatment groups in the community clinicians should address patient’s wishes for information, capture their interest in the group modality, and potentially offer a ‘trial’ session. Furthermore, they should make the group location and time as accessible as possible and create a moderately sized group of six to eight patients. In these groups, mutual respect, feelings of safety and encouragement appear essential to make patients feel they can benefit from attendance.

## Background

Treatment groups for psychiatric patients are routinely provided in community settings, in a variety of treatment modalities and for different diagnostic groups. Because of their cost-effectiveness and therapeutic advantages, they are recommended as a treatment for different groups of psychiatric patients [1–2].

Non-attendance (no-show or drop-out) to these groups has been a long-standing problem [3–4]. Rates of non-attendance have been reported between 19.7% [5] to 35% [6].

In some groups, non-attendance has been found to worsen patients treatment outcomes, (e.g. relapse in Alcoholics Anonymous) [7]. Other studies have found that non-attendance has a negative effect on the therapeutic group processes and group cohesion, impacting on the treatment for other patients who do attend [8–10]. As the resources are not utilised appropriately, non-attendance in groups has also been found to have negative financial ramifications for service providers [11].

Research into attendance has been criticised for its aims and methodology [12]. Although there are a wide number of treatment modalities for different diagnostic groups, studies investigating group attendance are often limited to the specific contexts of the groups under inquiry. These studies have rarely considered the provision of treatment groups in the community across modalities and specific diagnostic categories [12].

Furthermore, the literature has generally focused on identifying socio-demographic or clinical patient characteristics as predictor variables (e.g. age, ethnicity, diagnosis), [12–16] with the aim of improving selection or excluding non-ideal patients from group therapy [10]. Such an approach that focuses on improving selection ignores the structural or institutional barriers that prevent some from attending. Further, such studies have produced rather inconsistent findings [17] and found only small differences in such patient characteristics between attenders and non-attenders [18]. There is a growing recognition that there are relatively few socio-demographic or clinical patient characteristics that will predict attendance and that motivation for attendance intersects with the members of the group and the therapist [10]. Therefore it is recommended that research should go beyond looking into personal characteristics, and seek to understand ‘why’ patients do, or do not attend CMHGs, rather than ‘who’ does, or does not attend [10].

Additionally, studies that have attempted to identify patient characteristics as predictor variables have often done this via quantitative measures [10][14] thus potentially missing the unique and multifaceted motives as to why patients may, or may not attend groups [15]. Studies using qualitative methods have either focused on individualised therapy attendance [15], or used patients’ perspectives as a comparator to therapists’ perspectives [16–17]. This limits such findings as patients have not been studied in their own right [18]. A better understanding of facilitating and hindering factors from the patient perspective may help to improve attendance rates in treatment groups, and one may assume that there are some aspects that apply across different groups and treatments [19– 20].

Against this background, we explored the experiences of patients with serious mental illness who had attended, or had been invited to attend, different treatment groups in the community. We focused on the reasons for attending and not attending, to understand the facilitators and barriers to attendance.

## Methods

### Design

This study purposively selected qualitative interview data collected as part of three different studies that had investigated group treatment; A trial on Body Psychotherapy (BPT)[19–20], an observational study on Cognitive Behavioural Therapy (CBT)[21] and a feasibility trial on Music Therapy (MT) [22].

The first study aimed to evaluate the clinical effectiveness of BPT in the treatment of negative symptoms of schizophrenia. A full description of the study and its findings can be found elsewhere [19–20]. The multi-centre trial randomly allocated patients with a diagnosis of schizophrenia to a BPT or Pilates group. Patient’s capacity to consent was assessed by their referring clinician. The groups were run twice a week for ten weeks, with each session lasting ninety minutes. On completion of the groups, patients were interviewed about their experiences.

The second study aimed to identify and link group interactions and group experiences with outcomes [21]. As part of this study, a CBT group for people with psychosis was delivered. This group ran for ten weeks and consisted of fourteen sessions. Patient’s capacity to consent was assessed by their referring clinician and patients were interviewed post-intervention about their experience.

The third study was designed to develop best practice guidelines and examine the feasibility of a randomised controlled trial in group MT using songwriting with patients with long-term depression [22]. In preparation for the trial, individual semi-structured interviews were conducted with patients who had a diagnosis of depression. Patient’s capacity to consent was assessed by their referring clinician. In addition to this the capacity checklist tool developed by the British Psychological Society was administered before the interview [23]. Patients were asked questions relating to their past experiences of group therapies in the community.

### Sample

The interview data collected from the three studies were combined for analysis for two reasons. Firstly, the studies interviewed patients with experiences of a variety of treatment groups in the community, allowing the study to focus on group factors in general. Secondly, the interview topic guides used in these interviews all encouraged patients to discuss their lived experience of attending these groups, including motivations for attendance and drop out. The topic guide used in the MT study explicitly asked questions about attendance e.g. ‘why would patients want to attend or stop attending groups?’ whereas the topic guides used in the BPT and CBT studies were more implicit and asked questions regarding experiences of attending the groups e.g. ‘how did you find attending the group?’ and patients were encouraged to discuss facilitators and barriers.

Data were eligible for inclusion in this analysis if it met the following criteria:

- Was provided by someone aged 18 years or above
- The interviewee had given informed consent
- The interviewee had a confirmed diagnosis of schizophrenia (ICD-10 F2 spectrum, BPT and CBT study), depression (ICD-10 F3 spectrum, MT study) or anxiety disorder (ICD-10 F4 spectrum, MT study)
- The transcript had information relating to attending treatment groups in the community.

Ninety-eight transcripts of patient interviews were screened for analysis from the three studies. Sixty-seven transcripts from the dataset were eligible and analysed for this study. (Three transcripts were unavailable for analysis; twenty-eight transcripts were from patients who took part in a control group; Pilates. Therefore they did not have any reference to a mental health treatment group.) Transcripts where the patient had no experience of attending a treatment group in the community, but had previously been invited or spoke about future motivations to attend, were included.

Descriptions of ethical approvals and data collection procedures can be found in the original publications of these studies [19][21][22].

### Analysis

To manage the material, relevant sections of the transcripts, i.e. sections that referred to attendance or non-attendance, were extracted from the original transcript and exported into NVivo [24] and analysed using this software. During extraction of data from original transcripts we took a cautious approach and included any section referring to attendance or motivations. The framework method [25] is a form of qualitative analysis that uses a matrix output to organise large datasets to identify commonalities and differences within the data to draw descriptive and/or explanatory conclusions clustered around themes [26]. This analytic procedure was particularly useful for this study to help manage and organise the large amount of data. It also provided the opportunity to compare and contrast across different typologies, e.g. modality of groups.

To create the coding framework the first author (MD) open coded a random selection of transcripts (n = 10), while the second author (PM) coded five transcripts also selected at random. Initial categories were discussed by the two analysts, and early codes and categories that could be applied to all subsequent transcripts were decided upon. This initial ‘analytic framework’ went through many iterations as novel codes and categories were produced through the analysis process, and as discussions continued about the meaning of the findings between the analysts. A framework matrix was created once the analytic framework was finalised and data was charted into it [28].

Summarising the data in the matrix allowed the findings to be presented and discussed with all authors, the wider research group of the authors, as well as a patient and carer group (Service User and Carer Advisory Group on Research, run by City University of London and East London NHS Foundation Trust).

### Background of authors

The authors are qualified in psychology, psychiatry and music therapy and all work within academia. They all have experience of developing and/or delivering group treatments for patients with mental disorders.

## Results

### Participants

Sixty-seven interview transcripts were analysed for this study. Forty-nine patients were male, and sixteen female, (the gender of two patients was not recorded). The mean age of the patients was 41 (range 21–65). Fifty-five patients were unemployed, six were in employment, and one was in education. Employment statuses of five patients were not recorded. Fifty-two patients had a diagnosis of schizophrenia, twelve had a diagnosis of major depressive episode, and one patient had a diagnosis of anxiety. Two patient diagnoses were not recorded. As the interviews were conducted in the United Kingdom the patients had a wide variety of ethnic backgrounds.

Forty-six interview transcripts came from Study 1, eight transcripts came from Study 2 and thirteen from Study 3. Participants from these studies had experiences of different forms of treatment groups: BPT (68.7%); CBT (11.9%); general mental health support groups (GT) (9%), MT (6%) and no experience of attending group treatment (4.5%).

### Themes

Themes were ordered into the two groups of barriers and facilitators as per the aim of the study. Five themes were identified as facilitating factors and four as hindering factors. The factors facilitating attendance are presented first. Verbatim patient quotes have been provided alongside the analysis, with quotes accompanied by the type of group the patient had experience. Anonymized quotations can also be found in the S1 File.

### Facilitating attendance

#### Opportunity for autonomy

The autonomy to make choices on an ongoing basis was seen as key to encouraging attendance. When referred to a group, patients did not want to feel coerced by their clinician to attend the sessions.

The offer of a ‘taster’ session was something that was suggested a number of times by patients’. Such sessions were thought to encourage attendance by reducing the fear and anxiety of what might be expected of attendees.

*“Let them try it out for the day; there’s no obligation*. *And if they enjoy [it]*, *maybe they can sign up to take part in the group session…the option should be there for people to try it*.*” (P5*, *GT)*.

Even when people were attending group sessions, they enjoyed the ability to engage or disengage in the group process at will. This encouraged continuous attendance as it made the patients feel that their differences and needs were acknowledged and catered for in the groups. Many felt socially anxious about attending a group, so the option to attend without any expectation of contribution was seen as empowering.

*“I felt my anxiety level because I didn’t want to talk to people*, *but they gave me the choice of being quiet, just to come and listen, or you could talk” (P3, MT)*

#### Self-acknowledging need and therapist encouragement

Many patients were motivated to attend because they acknowledged that they required further intervention and saw group therapy as the next step to help improve their mental health.

*“If you want to help yourself you’ve got to do these things”. (P11*, *GT)*

Although there was an acknowledgement that further help was required, patients often spoke about the need for encouragement from the therapist to sustain attendance. Patients often listed qualities of staff that verbally and actively encouraged them (e.g. reassurance throughout the therapy and calling patients to confirm their attendance) as professional and non-judgemental.

*“She [therapist] usually says that the day that we’re not feeling on top of the world and if it’s the day of the therapy*, *that’s the day that we need it the most and we should try to come in rather than not attend.” (P4 GT)*

#### Optimal group format and safe environment

Patients had a preference for attending moderately sized groups of around six to eight people.

*“In my experience*, *no bigger than six or eight people because I think once you’ve gone above six to eight people you lose that interaction.” (P5, GT)*

Some patients seemed to be motivated to attend by having acquaintances that were also attending the same group, whereas complete anonymity motivated other patients.

*“These groups are anonymous. You don’t know anyone that you’re going to be in the group with. So you shouldn’t have a preconception of being felt as if you’re being judged or looked down on… nobody knows me. I don’t know them” (P5*, *GT)*

Additionally, patients preferred to attend groups that were welcoming, and that felt safe. Patient’s expectations of a safe place included an environment that was non-judgemental, confidential and where therapists and other patients respected autonomy in participation. Patients found that groups with such characteristics encouraged attendance as it allowed them to build gradual trust, therefore enabling attendants to share problems, listen to, be heard, and to work together.

*“They respected me and the way they talked to me*, *and the way they behaved. There was no abuse, there was no foul language, and there was no one being rude [it was] a safe place to share different ideas.” (P75, BPT)*

#### Interest in content and enjoyment

A factor that encouraged attendance for people who had attended arts therapies (MT and BPT) or who had no experience attending groups was whether or not they were interested in the type of group that was provided.

Even though a few patients were willing to ‘*try anything that might help*’ (*P5 GT*), others wanted their interest to be piqued. Enjoyment played a big part in this, with many referencing how personal enjoyment motivated attendance, or would motivate them in the future to attend.

*“If I got enjoyment out of it*, *I think that’s the most important thing for me*, *to go to something is to get enjoyment out of it*, *is something you look forward to*, *‘I can’t wait until next week*, *I want to go again*, *I enjoyed myself’*. *And if you*, *a person doesn’t get enjoyment out of it …they will think*, *well what is the point of me going all that way*?*” (P2*, *No experience)*.

When asked why they attended BPT *“There was music*, *some dancing*, *and I’d say overall a good atmosphere as well” (P63*, *BPT)*.

For some patients who attended arts therapy groups (MT and BPT) the enjoyment of the group reminded them of their past hobbies. These groups attracted patients who had mutual interests. And when enjoying themselves some patients did not feel like they were in a mental health group. This enabled reflection on thoughts and feelings beyond those limited to their mental illness.

*“It’s a great distraction from*, *thinking about yourself all the time…it’s very gentle…it's lots of fun;… it’s a great way of looking at feelings in a very gentle kind of way*, *through just tapping out rhythms” (P10*, *MT)*.

#### Actual / expected benefits of attendance

The potential benefits patients expected to gain from attendance ranged in a number of different ways but could be categorised into psychological, economic and social benefits.

Patients expect to psychologically benefit from attending a group.

*“for anyone who’s attending … they want to see at the end of it I suppose some kind of progress where it’s helped them… it would be a form of helping them maybe get a bit of relief while they was in that group to go and get away from their mental health problems they may be suffering and enjoy their self*, *take their mind off of whatever problems they may have. I think that would be the biggest thing that would help me, if I could just take my mind off of things for a while.” (P2, No experience)*

The psychological benefits patients gained from groups were widely varied. Patients found that by attending they learnt skills on how to cope with their mental illness and the associated symptoms. Some patients used groups as a way to break the routine of their lives and to provide structure to their daily living. A less common benefit that was mentioned was that the groups helped patients to develop their self-esteem and confidence.

*“I had this perception of myself that I didn’t know how to effectively communicate and so a lot of people in the group demonstrated to me that I was effective in communicating*, *it was a confidence booster” (P1*, *CBT)*.

Financial benefits were cited less frequently, but some interviewees attended the group sessions primarily because of the monetary incentives that were on offer. A few patients, who had either avoided attending groups or when attended had been paid, provided with lunch and transportation to and from the group (study 1) said they appreciated this and that they would not attend any other group that does not have these incentives.

*“As long as I get paid for it (attendance) I’m happy… [otherwise] I would not attend” (P13*, *No experience)*

Many patients reported benefiting socially from group attendance as such groups provided relief to the social isolation they were experiencing. It also presented an opportunity for them to meet people they could relate to, learn from and form friendships with.

*“I liked the group because I liked meeting other…likeminded people in the group who might have schizophrenia-like me” (P22*, *BPT)*

Some patients also spoke about how through attending groups they hoped to expand their support networks.

*“We could meet up*, *have a cup of tea and we can talk and organise a support network*, *like if one of us goes into the hospital*, *then one of us could go in and check that he’s ok” (P33*, *BPT)*.

The conversations people had before and after a group session seemed to be an important element to building rapport with other members. Similarly, the group processes (e.g. icebreakers) seemed to connect people by breaking down the barriers between people. Understanding that there was a shared experience of mental health issues and the comfort in this familiarity made people feel less alone and was a motivator for attendance.

*“I don’t get out to meet people*, *so this is a good chance for me to come to a group and just sit in and try to participate as much as I can” (P16, CBT)*

*“Sometimes it can be scary as hell hearing voices*, *and that is so good to see likeminded people talking about their experiences*, *talking about their meds and that” (P33*, *BPT)*.

### Barriers to attendance

#### Not being sufficiently informed

When patients felt they did not receive adequate information from their referring clinician about treatment groups, they often did not attend the group on offer. Furthermore, when patients were informed about ongoing treatment groups in the community, they were often given information in ways that were sub-optimal.

Discussing information sheets a patient quoted *“I don’t think bombarding us with the whole*, *full programme because sometimes that can be daunting…I know that I get confused if I have to read too much*, *and I forget what I read in the beginning by the time I reach the end*. *So it gets lost” (P4*, *GT)*

Patients recommended presenting the information about the groups using lay terms and using different mediums.

*“The information I would want would be either a text or an email or whatever the case may be and well about what the group was about” (P2*, *No experience)*.

However, opinion diverged on how much information patients thought were appropriate to receive pre-intervention. Some patients particularly appreciated full disclosure of information on the nature, purpose and structure of the group so that they could make an informed decision as to whether it was suitable for them.

*“Just covering all the bases really and making it interesting…just as much information as you can” (P12*, *GT)*.

Conversely, others felt that too much information could be ‘*daunting and little is better than more*’ (*P4*, *GT*).

Additionally, some patients highlighted that there was a discrepancy between the information clinicians provided (e.g. location and time), and the type of information they wanted to receive. Patients expected information on ‘*what the group was about*, *what it entailed and what the group did and the kinds of people that may be in the group*.’ *(P2*, *No experience)*

When information giving was congruent with patient needs (available, adequate, accessible and interesting), then it was seen as a motivator to attendance, rather than a barrier.

#### Concerns about social interactions and the unknown

Patients reported being self-conscious around new people and discussed concerns that they may not be able to express themselves properly or be misunderstood. Therefore, the idea of meeting new people and being expected to share feelings made them nervous.

*“I find it difficult to talk*. *I’ve got to ignore that machine [*internal voice*] otherwise I would start just losing my way*. *It’s all the inadequacies*, *self-esteem*, *have I said the right thing or the wrong thing and caring too much about what other people think*. *I was scared of going*. *Saying the wrong thing*, *people judging you*.*” (P11*, *GT)*

Patients’ concerns about social interactions were often related to previous bad experiences they had in groups. This may have had an impact on whether they felt they could trust other patients in groups. Patients who had previously attended groups highlighted that they could often be confrontational environments, where people are forced to share information with some members not respecting confidentiality.

*“People saying it outside of where it should be said” (P11*, *GT)*

Concerns about being judged or rejected, especially by other group members or group facilitators, were also widely mentioned during the interviews.

*“Maybe some people might feel as if they’re being judged or because obviously anything to do with the mind or the mental aspect*, *there’s a negative stigma, and people might not feel comfortable coming and talking about their experiences… I haven’t told anyone that I’m doing CBT” (P5, CBT)*

And there were some examples to suggest that some patients held negative views of others with the same diagnosis, and this was a reason why people did not attend.

*“I have stopped mixing with schizophrenics because the last schizophrenic I mixed with was just nasty like towards trying to be friends. So I just said no way am I mixing with schizophrenics again*, *just normal mates” (P67, BPT)*

#### Limited accessibility

Another barrier to attendance was the accessibility of the group. Accessibility was not restricted to just physical aspects but also included the requirement to meet their psychological or cultural needs.

Patients avoided attending groups that were held in inconvenient locations that had limited public transport links or required several public transport changes.

*“First of all how to get there*? *It should be within a reasonable area that you can get without too much difficulty… it has to be something that I can reach” (P4*, *GT)*.

This was particularly significant for those patients who were in employment (full time or volunteering) as groups that were held at inconvenient times interfered with their work schedule.

*“If you have a job*, *you cannot attend…unfortunately*, *I think the timing is the biggest problem” (P6*, *CBT)*.

Some patients highlighted that groups should take their diagnosis into consideration. For example, as a result of depression and anxiety patients reported feeling uncomfortable in large groups. Those taking medication felt that the side effects could lead to struggling to wake up in the mornings or lacking the motivation to attend.

*“I’ve got a sleep problem*, *and I tend to sleep most of the day and because I don’t want to wake up and go out” (P9, GT)*

Some patients highlighted that they did not attend certain groups because the group format (e.g. mixed gender groups) or content (e.g. music therapy) was not in line with their cultural and religious preferences.

*“Because they were men and I’m not allowed to speak to men*, *it’s against my religion” (P27*, *BPT)*.

#### Negative group dynamics

The dynamic of a treatment group was widely referenced as a factor that influenced patient’s decision to drop out. Patients who valued attending the groups found it demoralising and devaluing of the service when they perceived other attendees as not taking the group seriously (e.g. not attending, arriving late and using mobile phones during sessions).

*“When I’ve had group therapy*, *people arriving late, what’s the word I’m looking for? Well, people think it’s there for a laugh. When I go into things like this I go into it very seriously …I’ve got to give you 100%” (P8, GT)*

Even though patients acknowledged that it might be hard for some attendees to engage in the group, ultimately they found this to have a negative impact on the group. Actively engaged patients felt like the other members were not contributing to the group making the group feel arduous.

*“Some of them just don’t say a word*, *and it just gets long and laborious, and you feel like telling them that, say something.” (P4, GT)*

On the other hand, passive group members expressed difficulty when other group members dominated the group or appeared to be unaware of others’ needs.

*“People do get so engrossed with themselves that they’re not thinking about other members of the group if they can’t cope” (P8*, *GT)*.

Patients expected there to be different types of people within the groups. However, when these differences were too diverse (e.g. age differences and mixed diagnosis groups) they found that they could not relate to each other and could not form trusting relationships.

*“I found that most younger people did not take the group seriously. I think as you get older you tend to realise and understand (…)*, *what’s going on and it’s there for us. But when you’re young, you don’t understand that, so you think the world owes you something.” (P4, GT)*

Further, patients not only viewed the facilitator as someone who has the power to create and maintain a positive group dynamic but also as someone whose presence affected the group dynamic. Patients dropped out of groups when they felt that the facilitator could not handle the group dynamics properly, was unprofessional, inattentive and someone who did not explore issues properly.

*“I’ve been let down sometimes… either they’re [therapists] not handling it [sessions] properly*, *or they’re not exploring stuff properly and just allowing the group to go wherever they want to go” (P9*, *GT)*.

Non-attendance of other group members had a negative impact on the group dynamic. Many patients had experience of attending a group session where there were only two or three other group members. This meant that there was less opportunity to build group cohesion, cultivate relationships and engage in “*banter*” (*P4*, *GT*).

Many attended groups with the objective to make friends, or at least to socialise, and to have smaller than expected group sizes restricted their ability to do this.

In groups that could replace dropouts with new members, there was a feeling that it was difficult to build relationships with a constant influx of new people. This was especially salient for talking group therapies (CBT) which required members to repeat introductions or tell the same stories over again for newly arrived members which some found repetitive and tiring.

*“when a new member comes into the group*, *you’re repeating yourself so everyone that’s been there maybe a month before you has heard you say that seven or eight times so it can be, …oh yawning, I’ve heard this before.” (P20, CBT)*

Conversely, some patients felt there was a need for a quicker mechanism to fill the spaces that were made available due to non-attendance, to maximise the effect of the group and prevent the negative consequences discussed above.

*“If they’re going to leave*, *then that seat should be filled sooner rather than later because it’s*, *now that there’s only three of us*, *this week only two of us attended*, *so then that depletes you*.”(P4, GT).

## Discussion

### Main findings

The results suggest that patients are motivated to attend when they personally feel a need for the treatment, they are interested in the group on offer and satisfied with the information they received about the group. Furthermore, patients are motivated when they are given the opportunity for autonomy (e.g. through a trial session), when the group has an optimal size and is a safe environment, and when they feel they can benefit from attending.

Patients’ attendance was hindered when they were not satisfied with the information they received about the group, the group on offer was not accessible, or patients were concerned about the social interactions in the group, and they perceived or predicted negative group dynamics.

Insufficient information was a barrier to attendance, whilst on the other hand, appropriate information helped to increase attendance. Although some of the findings were to be expected, and reflect findings from previous studies, this qualitative analysis draws together a multitude of factors that hinder or help group treatment planning and delivery. The findings, therefore, suggest simple and practical considerations for the design and implementation of groups which may help improve attendance.

### Strengths and limitations

To our knowledge, this is the first contemporary qualitative study, which has investigated the facilitators and barriers to group attendance from the perspective of patients. This paper has gone beyond identifying patient characteristics as predictors of attendance and has explored the ‘lived experience’ of patients, thereby highlighting the wider social and structural barriers that impact on service delivery. The analysis also made use of a relatively large sample size of patients with a variety of experiences of group treatments.

Adopting the framework method to analyse the data has allowed us to gain a deeper understanding of the reasons behind attendance and non-attendance and enabled us to manage and analyse the large dataset (67 individual interview transcripts). This has resulted in the exploration of a broad range of views. As a result, the findings have some actionable implications for the design and implementation of treatment groups in the community.

The study also has limitations. The data came from three studies and from interviews that were conducted at different time points, post-intervention (Study 1 & 2) and pre-intervention (Study 3), and produced from three different topic guides. This difference in data collection time point and topic guides could have influenced what was said and the meaning of key concepts.

Furthermore, it could be argued that the available sample was biased by the fact that non-attenders to therapy would also be less likely to participate in the research interviews. However, the original studies actively tried to recruit non-attenders and dropouts, offering financial incentives to motivate participation.

Another limitation was that we could not ascertain how frequently patients attended the treatment groups under investigation, as data was not available. Additionally, the findings focus on facilitators and barriers to groups as a whole, and not on the theory specific mechanisms of change that are theorised to be at work. We could not establish from this dataset whether theory specific elements of each group type were implicated in reasons for attending or drop out.

### Comparison with literature

Research into attendance generally has distinguished reasons for attendance and non-attendance into reasons that are preventable (e.g. providing adequate information) and things that are unavoidable (e.g. self-acknowledging need)[27]. Although the findings from this study could also be categorised in such a way, this overlooks the ease with which some factors could be prevented through better planning in comparison to others.

Adequate preparation is a theme that was highlighted by Yalom [28] and Bernard et al. [29], and it could be argued that some of the barriers identified here could be easily overcome by adequate preparation. Factors such as providing sufficient information or accessible venues were seen to be key as to how successful a group was and are relatively easy to implement.

However, therapists organising groups face numerous demands on their time and resources and finding the time to juggle the numerous tasks when organising and facilitating a group has been shown to be difficult [27]. Some of these challenges include supporting new members, encouraging patients to adhere to the treatment, trying to prevent drop out and dealing with transdiagnostic groups [27]. Our research highlights the importance of factors that are preventable through provision by statutory services and adequate preparation.

The findings from this study also show how groups are social spaces that have the potential to produce non-specific treatment outcomes [30]. Social benefits were an often-cited motivator for attendance in the interviews, and this supports the previous literature about the importance of group format [31], but equally shows how negative group dynamics can have detrimental consequences [32].

The findings of this study are similar to that of Barret et al.,[4] who suggest that the reasons for attendance may not be parallel to those for non-attendance. The findings presented here demonstrate that there were unique and separate reasons cited for facilitators and barriers for group attendance. This means that there are different pathways to increasing attendance, through both reducing drop out through facilitation and removing barriers to engage and encourage usual ‘non-attenders’. Careful consideration therefore needs to be given by clinicians in striking a balance between both investing in facilitators and removing barriers. It is difficult to say which strategy will yield better results but findings here suggest both aspects may be important to improve attendance.

The only factor that was both a barrier and facilitator to group attendance was whether patients felt well or poorly informed. The divergence in opinion by the patients on how information should be presented and how much should be provided has indicated that there is no “one size fits all” approach and that information should be tailored to patients’ wishes. Although this may be a time-consuming process the finding that the provision of adequate information motivated, and deficient provision of information hindered attendance, demonstrates the importance of tailored communication.

The findings from this study also fit with theories of motivation [33]. The self-determination theory of motivation suggests that circumstances promoting autonomy empower people and lead to improved motivation [34]. Previous studies investigating effective strategies to improve attendance have found that offering patients choice was the single most effective strategy [35]. Whilst it might be unrealistic to give every patient the autonomy to determine every aspect of therapy (especially within group formats), making small concessions to empower patients (such as allowing them to engage in group activities at will as was suggested within the data), may help to increase attendance rates. However, some suggestions made by patients, e.g. taster sessions, although popular, may be difficult, if not impossible, to implement in practice being costly in terms of both time and resources. Similarly, the idea of engaging in the group activities at will may be detrimental to the group process. Not all suggestions made by patients are actionable or even pragmatic within mental health services, and thus this reiterates the importance of having an overview of barriers and facilitators and categorising them in terms of ease of implementation, not just how preventable or unavoidable they are [29].

Future treatment groups could offer patients information using different sources (written, audio-visual and a ‘taster session’).This study also underscores the importance of considering impact upon group dynamic when recruiting a new patient into a group.

When refering patients into groups clinicians could ask whether patients have cultural requirements or preference for single gender community groups. Therapists might ascertain preferences for contact (such as reminders) in advance of the group and set clear and supportive expectations as to how to manage times when attendance is compromised. Patients could be provided with incentives such as travel cover, lunch or money to encourage attendance. The groups could be moderately sized (six to eight people), and the venue could be in a location that is accessible (transport links) to patients.

Time could be provided before, during and after the groups for patients to socialise. During the sessions, setting ground rules could help build mutual respect for the group and encouraging equal contribution could promote every patient to benefit from the group.

## Conclusion

The findings of this study are largely in agreement with the previous literature. This qualitative analysis has drawn together a number of barriers and facilitators that will inform group treatment planning and delivery. There are unique and separate facilitators for and barriers to group attendance, meaning that different strategies may have to be employed to improve attendance.

Furthermore, the findings highlighted that groups are social spaces and that through adequate preparation, e.g. appropriate information giving and venue choice, attendance may be improved.

Future research should seek to test to what extent the suggestions based on this study can be implemented in routine practice and to what extent they lead to actually improved attendance rates.

## Supporting information

S1 File Relevant anonymized quotations. (DOCX)
