# Supplementary material for: Why do psychiatric patients attend or not attend treatment groups in the community: A qualitative study
Source: PLoS One. 2018 Dec 13;13(12):e0208448. doi: 10.1371/journal.pone.0208448 (PMC6292613; doi:10.1371/journal.pone.0208448)
Supplement: S1 File — (DOCX) [file pone.0208448.s001.docx]

**Relevant anonymized quotations**

| **Opportunity for autonomy** | *“Let them try it out for the day; there’s no obligation. And if they enjoy [it], maybe they can sign up to take part in the group session…the option should be there for people to try it.” (P5, GT).*  *“I felt my anxiety level because I didn’t want to talk to people, but they gave me the choice of being quiet, just to come and listen, or you could talk” (P3, MT)* |
| --- | --- |
| **Self-acknowledging need and therapist encouragement** | *“If you want to help yourself you’ve got to do these things”. (P11, GT)*  *“She [therapist] usually says that the day that we’re not feeling on top of the world and if it’s the day of the therapy, that’s the day that we need it the most and we should try to come in rather than not attend.” (P4 GT)* |
| **Optimal group format and safe environment** | *“In my experience, no bigger than six or eight people because I think once you’ve gone above six to eight people you lose that interaction.” (P5, GT)*  *“These groups are anonymous. You don’t know anyone that you’re going to be in the group with. So you shouldn’t have a preconception of being felt as if you’re being judged or looked down on… nobody knows me. I don’t know them” (P5, GT)*  *“They respected me and the way they talked to me, and the way they behaved. There was no abuse, there was no foul language, and there was no one being rude [it was] a safe place to share different ideas.” (P75, BPT)* |
| **Interest in content and enjoyment** | ‘*try anything that might help*’ (*P5 GT*),  *“If I got enjoyment out of it, I think that’s the most important thing for me, to go to something is to get enjoyment out of it, is something you look forward to, ‘I can’t wait until next week, I want to go again, I enjoyed myself’. And if you, a person doesn’t get enjoyment out of it …they will think, well what is the point of me going all that way?” (P2, No experience).*  *“There was music, some dancing, and I’d say overall a good atmosphere as well” (P63, BPT).*  *“It’s a great distraction from, thinking about yourself all the time…it’s very gentle…it's lots of fun;… it’s a great way of looking at feelings in a very gentle kind of way, through just tapping out rhythms” (P10, MT).* |
| **Actual / expected benefits of attendance** | *“for anyone who’s attending … they want to see at the end of it I suppose some kind of progress where it’s helped them… it would be a form of helping them maybe get a bit of relief while they was in that group to go and get away from their mental health problems they may be suffering and enjoy their self, take their mind off of whatever problems they may have. I think that would be the biggest thing that would help me, if I could just take my mind off of things for a while.” (P2, No experience)*  *“I had this perception of myself that I didn’t know how to effectively communicate and so a lot of people in the group demonstrated to me that I was effective in communicating, it was a confidence booster” (P1, CBT).*  *“As long as I get paid for it (attendance) I’m happy… [otherwise] I would not attend” (P13, No experience)*  *“I liked the group because I liked meeting other…likeminded people in the group who might have schizophrenia-like me” (P22, BPT)*  *“We could meet up, have a cup of tea and we can talk and organise a support network, like if one of us goes into the hospital, then one of us could go in and check that he’s ok” (P33, BPT).*  *“I don’t get out to meet people, so this is a good chance for me to come to a group and just sit in and try to participate as much as I can” (P16, CBT )*  *“Sometimes it can be scary as hell hearing voices, and that is so good to see likeminded people talking about their experiences, talking about their meds and that” (P33, BPT).* |
| **Not being sufficiently informed** | *“I don’t think bombarding us with the whole, full programme because sometimes that can be daunting…I know that I get confused if I have to read too much, and I forget what I read in the beginning by the time I reach the end. So it gets lost” (P4, GT)*  *“The information I would want would be either a text or an email or whatever the case may be and well about what the group was about ” (P2, No experience).*  *“Just covering all the bases really and making it interesting…just as much information as you can” (P12, GT).*  Too much information could be ‘*daunting and little is better than more*’ (*P4, GT*).    Patients expected information on ‘*what the group was about, what it entailed and what the group did and the kinds of people that may be in the group*.’ *(P2, No experience)* |
| **Concerns about social interactions and the unknown** | *“I find it difficult to talk. I’ve got to ignore that machine [*internal voice*] otherwise I would start just losing my way. It’s all the inadequacies, self-esteem, have I said the right thing or the wrong thing and caring too much about what other people think. I was scared of going. Saying the wrong thing, people judging you.” (P11, GT)*  *“People saying it outside of where it should be said” (P11, GT)*  *“Maybe some people might feel as if they’re being judged or because obviously anything to do with the mind or the mental aspect, there’s a negative stigma, and people might not feel comfortable coming and talking about their experiences… I haven’t told anyone that I’m doing CBT” (P5, CBT)*  *“I have stopped mixing with schizophrenics because the last schizophrenic I mixed with was just nasty like towards trying to be friends. So I just said no way am I mixing with schizophrenics again, just normal mates” (P67, BPT)* |
| **Limited accessibility** | *“First of all how to get there? It should be within a reasonable area that you can get without too much difficulty… it has to be something that I can reach” (P4, GT).*    *“If you have a job, you cannot attend…unfortunately, I think the timing is the biggest problem” (P6, CBT).*  *“I’ve got a sleep problem, and I tend to sleep most of the day and because I don’t want to wake up and go out” (P9, GT)*  *“Because they were men and I’m not allowed to speak to men, it’s against my religion” (P27, BPT).* |
| **Negative group dynamics** | *“When I’ve had group therapy, people arriving late, what’s the word I’m looking for? Well, people think it’s there for a laugh. When I go into things like this I go into it very seriously ...I’ve got to give you 100%” (P8, GT)*  *“Some of them just don’t say a word, and it just gets long and laborious, and you feel like telling them that, say something.” (P4, GT)*  *“People do get so engrossed with themselves that they’re not thinking about other members of the group if they can’t cope” (P8, GT).*  *“I found that most younger people did not take the group seriously. I think as you get older you tend to realise and understand (…), what’s going on and it’s there for us. But when you’re young, you don’t understand that, so you think the world owes you something.” (P4, GT)*  *“I’ve been let down sometimes… either they’re [therapists] not handling it [sessions] properly, or they’re not exploring stuff properly and just allowing the group to go wherever they want to go” (P9, GT).*  *“when a new member comes into the group, you’re repeating yourself so everyone that’s been there maybe a month before you has heard you say that seven or eight times so it can be, …oh yawning, I’ve heard this before.”* *(P20, CBT)*  *“If they’re going to leave, then that seat should be filled sooner rather than later because it’s, now that there’s only three of us, this week only two of us attended, so then that depletes you*.”(P4, GT). |
